# Loss of heterozygosity impacts MHC expression on the immune microenvironment in CDK12-mutated prostate cancer

**DOI:** 10.1186/s13039-024-00680-6

**Published:** 2024-05-04

**Authors:** William Lautert-Dutra, Camila M. Melo, Luiz P. Chaves, Cheryl Crozier, Fabiano P. Saggioro, Rodolfo B. dos Reis, Jane Bayani, Sandro L. Bonatto, Jeremy A. Squire

**Affiliations:** 1 Department of Genetics, Medical School of Ribeirao Preto, University of Sao Paulo - USP, Ribeirão Prêto, SP 14048-900 Brazil; 2 Diagnostic Development, Ontario Institute for Cancer Research, Toronto, ON Canada; 3 Department of Pathology, Ribeirao Preto Medical School, University of Sao Paulo - USP, Ribeirão Prêto, Brazil; 4 Division of Urology, Department of Surgery and Anatomy, Medical School of Ribeirao Preto, University of Sao Paulo - USP, Ribeirão Prêto, Brazil; 5 Laboratory Medicine and Pathology, University of Toronto, Toronto, ON Canada; 6 School of Health and Life Sciences, Pontifical Catholic University of Rio Grande Do Sul - PUCRS, Av. Ipiranga, 668, Porto Alegre, RS 90619-900 Brazil; 7 Department of Pathology and Molecular Medicine, Queen’s University, Kingston, ON K7L3N6 Canada

**Keywords:** Somatic chromosomal alterations, Copy Number Associations, Biomarkers, Immunotherapy, Transcriptomics, Public domain databases, Digital cytometry, Antigen presentation, Immune evasion, Adaptative immune system

## Abstract

**Background:**

In prostate cancer (PCa), well-established biomarkers such as MSI status, TMB high, and PDL1 expression serve as reliable indicators for favorable responses to immunotherapy. Recent studies have suggested a potential association between CDK12 mutations and immunotherapy response; however, the precise mechanisms through which CDK12 mutation may influence immune response remain unclear. A plausible explanation for immune evasion in this subset of *CDK12*-mutated PCa may be reduced MHC expression.

**Results:**

Using genomic data of *CDK12*-mutated PCa from 48 primary and 10 metastatic public domain samples and a retrospective cohort of 53 low-intermediate risk primary PCa, we investigated how variation in the expression of the MHC genes affected associated downstream pathways. We classified the patients based on gene expression quartiles of MHC-related genes and categorized the tumors into “High” and “Low” expression levels. *CDK12-*mutated tumors with higher MHC-expressed pathways were associated with the immune system and elevated *PD*-*L1*, *IDO1*, and *TIM3* expression. Consistent with an inflamed tumor microenvironment (TME) phenotype, digital cytometric analyses identified increased CD8 + T cells, B cells, γδ T cells, and M1 Macrophages in this group. In contrast, *CDK12*-mutated tumors with lower MHC expression exhibited features consistent with an immune cold TME phenotype and immunoediting. Significantly, low MHC expression was also associated with chromosome 6 loss of heterozygosity (LOH) affecting the entire *HLA* gene cluster. These LOH events were observed in both major clonal and minor subclonal populations of tumor cells. In our retrospective study of 53 primary PCa cases from this Institute, we found a 4% (2/53) prevalence of *CDK12* mutations, with the confirmation of this defect in one tumor through Sanger sequencing. In keeping with our analysis of public domain data this tumor exhibited low MHC expression at the RNA level. More extensive studies will be required to determine whether reduced *HLA* expression is generally associated with primary tumors or is a specific feature of *CDK12* mutated PCa.

**Conclusions:**

These data show that analysis of *CDK12* alteration, in the context of MHC expression levels, and LOH status may offer improved predictive value for outcomes in this potentially actionable genomic subgroup of PCa. In addition, these findings highlight the need to explore novel therapeutic strategies to enhance MHC expression in *CDK12*-defective PCa to improve immunotherapy responses.

**Supplementary Information:**

The online version contains supplementary material available at 10.1186/s13039-024-00680-6.

## Background

Treatment of advanced prostate cancer (PCa) remains a therapeutic challenge. Men with distant metastases at diagnosis have the poorest overall survival, with only 30% of patients surviving > 5 years [1]. For recurrent disease, acquired resistance to androgen deprivation therapy and chemotherapy remains a significant cause of death [2]. Immune-checkpoint blockade (ICB) therapies, such as PD-L1 inhibition, have shown only significant benefits in a minority of patients [3]. Thus, it is necessary to discover and characterize the genetic pathways and molecular signatures that could help predict more effective disease progression control in advanced PCa.

Tumor mutation burden affects the infiltration of immune cells in the tumor microenvironment (TME) [4]. Tumors with defective DNA damage repair (DDR) pathways, resulting in a high neoantigen load, are more suitable for immunotherapy [5]. The biallelic inactivation of Cyclin-dependent Kinase 12 (*CDK12*) prevents the formation of the CDK12/cyclin K complex and impairs the phosphorylation of the C-terminal heptapeptide of the RNA polymerase 2. This interference affects the transcription elongation, splicing, cleavage, and polyadenylation of a group of genes, including those crucial for DDR [6, 7]. This loss-of-function leads to DNA instability and genomic alterations and sensitizes cells to DNA damage agents. This susceptibility has been observed in different cancer models, including breast and ovarian carcinoma, as well as Ewing sarcoma [8–11]. Recently, a novel immune-active class of advanced PCa has been identified, characterized by a more aggressive phenotype, a high mutation burden derived from focal tandem duplication events, and elevated levels of inflammatory and immune cell infiltrates distinct from other defective molecular subtypes [12–14]. Studies have reported varying prevalence rates of *CDK12* mutations in PCa, typically ranging from 1 to 5% [12, 14, 15]. Searching public domain genomic databases for *CDK12* inactivating mutations in primary and metastatic PCa, represents a valuable approach to understanding the molecular pathways associated with immune evasion within this rare subtype.

Although this new subtype has been proposed as a predictive biomarker of treatment response to ICB in advanced PCa, many patients with *CDK12* alterations still fail to respond to ICB treatment. In a retrospective multi-center study, Antonarakis et al*.* reported that only 33% of the *CDK12*-altered advanced PCa patients had a prostate-specific antigen (PSA) response and an increased progression-free survival of 5.4 months when treated with anti-PD-1 therapy [16]. In another study, Schweizer et al*.* showed that of the 19 advanced patients who received ICB, 11 (59%) showed a response based on a decline in PSA, with two patients (11%) having a 100% PSA decline [17]. However, the molecular causes of either intrinsic or acquired ICB resistance in this distinct molecular subtype of PCa are poorly understood.

Tumor cells may develop various escape mechanisms that avoid recognition and destruction by the immune system. For example, the expression of checkpoint proteins (PD-1/PD-L1, CTLA-4, LAG3) by tumor cells can modulate the activity of immune infiltrate cells [18]. They may also develop intrinsic cancer-cell signaling (WNT/β-catenin) that can suppress infiltrating immune cells and increase pro-tumorigenic immune cell infiltration (Tregs, M2 Macrophages) in the TME [19, 20]. Another mechanism exploited by tumors to escape recognition by cytotoxic T cells (CD8 +) and antigen-presenting cells (APCs) is through loss of major histocompatibility complex class-I or class-II (MHC-I and MHC-II) [21].

Changes in MHC expression have been linked to tumor progression, poor prognosis, and reduced response to ICB in different malignancies [22, 23]. As classified by Garrido et al., the alteration in the MHC expression can be divided into two major mechanistic groups: tumors with “Soft” alterations are capable of recovering or upregulating MHC antigens after cytokine exposure (e.g., characterized as having regulatory abnormalities); whereas those with “Hard” alterations cannot recover MHC expression (e.g., characterized as having chromosomal alterations such as loss-of-heterozygosity (LOH)) [24]. During tumor evolution, the infiltration of cytotoxic lymphocytes eliminates highly immunogenic tumor clones, causing a selection of surviving cell populations that have acquired MHC alterations through either “Soft” or “Hard” mechanistic alterations [25]. Low expression of MHC-I and -II has been associated with poor prognosis and resistance to anti-CTLA-4 and anti-PD-1, respectively [26, 27]. However, in PCa, there is presently limited information on the role of MHC-I and MHC-II expression and ICB response and whether low expression of MHC could better predict the lack of response to ICB in *CDK12*-altered patients.

In PCa, *CDK12* inactivation is known to increase the immunogenicity of tumor cells, but the relationship between *CDK12* loss and MHC expression has not been investigated. Changes in MHC-I and -II expression are involved in tumor immune evasion in various types of cancer [25]. There is also evidence that higher expression of MHC genes can identify tumors likely to respond to ICB [22, 23]. However, the molecular and genomic mechanisms responsible for modulating MHC expression are poorly understood. Hence, we hypothesize that variation in the expression of the MHC genes could explain the variable responses to ICB in 40–66% of *CDK12* defective tumors [16, 17, 28]. Our initial transcriptomic analysis was based on 58 *CDK12* mutated PCa derived from large public-domain datasets. Our in silico analysis revealed that *CDK12* defective PCa tumors that express higher levels of MHC are characterized by immunomodulator pathway expression such as IFN-γ-response and cytotoxic activity genes. This subset also possesses an inflamed TME with increased presence of effector T cells. In contrast, the *CDK12* mutant tumors with lower MHC expression were associated with an immunologically cold TME. The impact of *MHC* expression on downstream pathways was validated using transcriptomic data from a 53-patient cohort from our Institute. Further investigation of public domain data showed that PCa with decreased MHC expression also exhibited chromosome 6 specific LOH of the *HLA* gene cluster and genomic loci associated with genes involved in antigen presentation. These genes are closely involved in activating MHC expression and the presentation of antigens, so their reduced expression may account for the failure of a subset of PCa with *CDK12* alteration to respond to immunotherapy. Collectively, these data suggest that the subset of PCa with *CDK12* alteration may have acquired chromosome 6 alterations such as LOH that reduce the expression of MHC, leading to an immune evasion phenotype.

## Methods

### Public domain databases

In this study, we examined public domain genomics databases comprising 488 primary PCa and 150 metastatic PCa. The CbioPortal for Cancer Genomics [29] was used to search primary (pPCa) and metastatic castration-resistant (mCRPC) prostate tumors with *CDK12* alterations with matched clinical information. Details of patient treatments prior to therapy are not provided. The classification of samples having *CDK12* loss-of-function was consistent with previous reports [7, 13–15]. CDK12 alteration group (CDK12-Mut) was defined by the presence of somatic alterations (non-synonymous mutations, deep deletions, and shallow deletions) in one or both CDK12 alleles*.* Only studies containing *CDK12*-mut samples with whole exome sequencing (WES) and RNA-seq data were selected in the Genotypes and Phenotypes (dbGaP) database and applied for access under project ID 29255 (Additional file 1: Fig. S1, Additional file 2: Table S1﻿).

### Transcriptomic analysis

For the pPCa cohort (TCGA-Prostate Adenocarcinoma, *n* = 48), we used recount2 [30] to download summarized experiments objects containing the transcription-level RNA-Seq abundance matrix [31]. For the mCRPC cohort (SU2C, *n* = 10), we downloaded SRA Paired-End (PE) reads using the SRAToolkit (https://github.com/ncbi/sra-tools). The quality of the raw reads were then measured using the FASTQC program (https://www.bioinformatics.babraham.ac.uk/projects/fastqc/). We quantified the transcripts using Salmon v1.6.0 directly in the human transcriptome [32]. The transcriptome index was built using the reference GRCh38 version of the human genome and transcriptome, downloaded from ENSEMBL [33] and GENCODE [34], following the manual instructions. We then used Tximport v1.22.0 (https://github.com/thelovelab/tximport) to import the transcription-level abundance and estimate raw counts derived from the quantification step. The count data normalization, expression levels, and differential gene expression (DEG) analysis for the pPCa and mCRPC samples were executed using DESeq2 v1.34.0 [35, 36]. We used clusterProfiler v4.2.1 [37] to implement equally over-representation (ORA) enrichment analysis and gene set enrichment analysis (GSEA) of the DEGs and the whole transcriptome profile, respectively, using the Gene Ontology (GO) and Molecular Signatures Database (MSigDB) [38–42].

The PanImmune Panel (NanoString Technologies Inc., Seattle, WA, USA) was used to profile the RNA derived from the FFPE samples from our 53-patient tumor cohort (see below). Raw expression data from the PanImmune Panel was loaded in nSolver software v4.0 (NanoString Technologies) to perform the quality control (QC analysis) and to build the transcript matrix for downstream analysis.

### MHC expression and patient classification

To test the biological variation related to MHC expression in pPCa and mCRPC with CDK12-mut tumors, we performed hierarchical clustering analysis using representative MHC genes. We observed two groups of *CDK12-*mut tumors based on MHC expression, as shown in Additional file 1: Fig. S1. Patients were classified based on gene expression quartiles and dichotomized expression levels below or above the first quartile for each gene; this classification was then used to generate the final logical values with respect to MHC status (‘High’ or ‘Low’ expressed) (Additional file 1: Fig. S1) [43, 44]. Our rationale for classifying cases as "MHC low" or "MHC high" is based on previous research demonstrating correlations between HLA gene expression and surface protein levels [45]. For the quartile quantification, the normalized expression values of the classical genes that composed each MHC class were used (e.g., MHC-I: *HLA-A*, *HLA-B*, *HLA-C*; MHC-II: *HLA-DPA1*, *HLA-DPB1*, *HLA-DQA1*, *HLA-DQB1*, *HLA-DQB2*, *HLA-DRA*, *HLA-DRB5*, *HLA-DRB6*) [44]. Samples were classified as having MHC ‘Low’ expression when at least one gene composing each class was expressed at a low level. In mCRPC *CDK12*-mut cases, all tumors classified as MHC-High presented similar high expression of both MHC-I and -II; thus, these tumors were identified as MHC High (henceforth, mCRPC *CDK12*-mut MHC High). Our retrospective cohort of low-intermediate risk pPCa derived from radical prostatectomies was used to validate our transcriptomic findings that were derived from public-domain samples.

### Genomic profile

We downloaded SRA Paired-End reads for both pPCa (n = 48) and mCRPC (n = 10) tumors and processed them as described above. The GRChg38 reference was sorted using SeqKit [46]. The final fastq files were then aligned to hg38 using bwa with a penalty for up to 3 mismatches per read [47, 48]. Sam files were converted to bam files and processed using samtools v1.16.1. (https://samtools.github.io). To determine whether any MHC expression differences were related to genomic alterations, we used the FACETS (Fraction and Allele-Specific Copy Number Estimated from Tumor Sequencing) algorithm [49]. This approach uses matched normal-tumor WES and provides mutant allele-specific copy-number homozygous/heterozygous deletions, chromosome-specific copy-number neutral LOH, allele-specific gain/amp in genomic loci associated with genes involved in antigen presentation (Additional file 3: Table S2). Reference and variant allele read counts were extracted from the bam file for common, polymorphic SNPs downloaded from dbSNP (GRCh38p7) using FACETS *snp*_*pileup* function (https://github.com/mskcc/facets/tree/master/inst/extcode) with a minimum threshold for mapping quality, the minimum threshold for the base quality, and minimum read depth of 15, 20, 20, respectively. The pre-processing followed the suggested recommendations from the manual, and genomic intervals of 150-250bp were used to avoid hyper-segmentation in high polymorphic neighborhood regions. Mutant allele-specific copy-number changes were declared when the points changes were greater than a pre-determined critical value (cval) of 100 compared to constant copy-number regions [50, 51].

### Digital cytometry

To investigate and quantify the immune cell composition in the TME of tumors having *CDK12-*mut, we used the bulk tissue gene expression profiles (GEP) from the RNA-seq data from both pPCa and mCRPC tissues with the digital cytometry resource CIBERSORTx [52–54]. This algorithm uses bulk tissue GEP, compares the data with prior knowledge of expression profiles from purified leukocytes, and estimates a tumor's relative immune abundance composition. We used the ‘signature matrix’ containing a validated leukocyte GEP of 22 human hematopoietic cell phenotypes, leukocyte gene signature matrix (LM22), to estimate the immune cell composition from the TME.

### Validation

To validate our transcriptomic findings and genomic analysis, we used a retrospective cohort of clinical intermediate pPCa derived from radical prostatectomies (*n* = 53) performed at the Faculty of Medicine of Ribeirao Preto (FMRP). All 53 samples included in the FMRP cohort were pPCa collected by radical prostatectomy following National Comprehensive Cancer Network (NCCN) clinical practice guidelines [55] in the Department of Surgery and Anatomy, Urology Division at Ribeirao Preto Medical School, Brazil, between 2007 and 2015 (Additional file 2: Table S1). According to the American College of Pathology, the smaller prostates were submitted in their entirety. For partial sampling in the setting of larger glands, we followed the protocol of submitting always whole grossly visible tumor (when identified), the tumor and associated periprostatic tissue and margins, along with the entire apical and bladder neck margins and the junction of each seminal vesicle with prostate proper. If there is no grossly visible tumor, a systematic sampling strategy was used that concerns submitting the posterior aspect of each transverse slice along with a mid-anterior block from each side, and the entire apical and bladder neck margins and the junction of each seminal vesicle with the prostate. The patients were classified according to the presence of biochemical recurrence (BCR), defined as PSA > 0.2 ng/ml within six months after radical prostatectomy. Patient outcome data were collected to the last follow-up date (Additional file 2: Table S1). This retrospective study was approved by the Ethics Committee in Research of Hospital of Ribeirão Preto, São Paulo, Brazil (HCRP) number CAAE 60032122.8.0000.5440 and the Ethics Board of the University of Toronto (Protocol: 00043323).

The DNA/RNA was isolated from tissues with tumor-rich areas previously marked by a pathologist (FPS) which represent the highest Gleason pattern. Sections were processed at the Ontario Institute for Cancer Research, Toronto, Canada (OICR) using a dual DNA and RNA extraction as previously described [56, 57]. Hematoxylin and eosin slides were prepared for all the Formalin-Fixed Paraffin-Embedded (FFPE) tissues. The percentage of tumor cells (range 70–95% tumor-rich) within each marked tumor-rich area was estimated and recorded. Adjacent slides for each tumor were prepared, and the same areas of interest were microdissected for RNA/DNA extraction.

The RNA profiling was performed according to the manufacturer’s instructions using mRNA PanImmune Panel (NanoString Technologies Inc., Seattle, WA, USA). Raw expression data from the PanImmune Panel was loaded in nSolver software v4.0 (NanoString Technologies) to perform the quality control (QC analysis) and to build the transcript matrix for downstream analysis. Because of the limited number of samples, patients were classified as having MHC ‘Low’ or “High” expression (including both MHC-I and -II) as previously described for MHC-I.

Raw DNA data was generated from the Oncomine Comprehensive Assay Plus panel (OCA-Plus, Thermo Fisher Scientific), which profiles 501 genes for single and multiple gene biomarkers. Library construction and sequencing were performed according to the manufacturer’s instructions (Thermo Fisher Scientific). The read sequence and processing were performed using the Ion Torrent platform. The data were mapped to the human genome hg19, indicated as the reference genome in the Ion Reporter software v5.18 (Thermo Fisher Scientific). Oncomine Comprehensive Plus—w2.3—DNA—Single Sample was used as analysis workflow for the OCA-Plus panel. The output regarding coverage, mean depth uniformity and alignment over the reference were used as references for quality assessment.

Formalin fixation causes the deamination of nucleotides producing base changes of C to T and G to A, which have been identified as a significant factor of low-frequency sequence artifacts not present in the original sample. The frequency of these artifacts is predicted to be present in all samples and with an allelic frequency below 5–10% [58]. To address the presence of false-positive changes derived from potential technical artifacts, annotated variant call format files providing all identified variants were filtered as follows. First, variants were filtered if they had not met the following criteria, (1) allele frequency VAF > 10%, (2) *p* < 0.0001, and (3) coverage > 350. Second, variants isolated from highly deaminated passed for an additional filter, which excluded variants below VAF > 15%, *p* < 0.0001, coverage > 350, and Phread 300. Additionally, variants were excluded if they were detected in all samples [59].

### PCR and Sanger sequencing

Two patients that showed evidence of *CDK12* mutation (Brazil-17 and Brazil-38) were subjected to PCR amplification and Sanger sequencing *to further confirm the presence of a CDK12 mutation in the tumor DNA.* As a negative control, we used DNA from one internal control breast cancer sample (Breast Control) and one sample with negative evidence of *CDK12* mutation (Brazil-14) as determined by the OCA-Plus panel. The primers and amplicons utilized are displayed in Additional file 4: Table S3.

We followed the protocol instructions for the PCR amplification using AmpliTaq Gold® 360 Master Mix from Applied Biosystems. For sample #17's CDK12 amplicons 1A and 1B, 75 ng of input DNA were used and 35 PCR cycles at 60 ºC were performed. For *CDK12* amplicon 2B from sample #38, due to low quality DNA, 100 ng of input DNA were used with 40 cycles at 60 ºC. The PCR products were subsequently purified using the ExoSAP-ITTM Express PCR Product Cleanup protocol from Applied Biosystems. Ten nanograms (10ng) purified PCR product were sent to The Centre for Applied Genomics (TCAG, The Hospital for Sick Children, Toronto, CA) sequencing facility for Sanger Sequencing (http://tcag.ca/facilities/dnaSequencingSynthesis.html). The sequencing files were loaded on the Thermo Fisher cloud (https://apps.thermofisher.com/apps/spa) and visualized the Next-Generation Confirmation app.

### Computational and statistical analysis

A GNU/Linux environment was used to perform quality control and quantify the raw reads to the human transcriptome. Subsequently, downstream analysis was performed in RStudio (R Foundation for Statistical Computing, R v4.1.2). Pearson Correlation was used to analyze the normalized expression levels (coef. level = 0.95). A gene was considered differentially expressed when log2 foldchange > 1 was expressed from the reference group with a *P*-adjusted value < 0.05. For the enrichment analysis, we used a cutoff value of 0.05.

## Results

### CDK12-mut MHC-I/-II high-expression tumors showed distinct transcriptome profiles with upregulation of IFN-γ-responsive genes and an inflamed TME

**Fig. 1:**
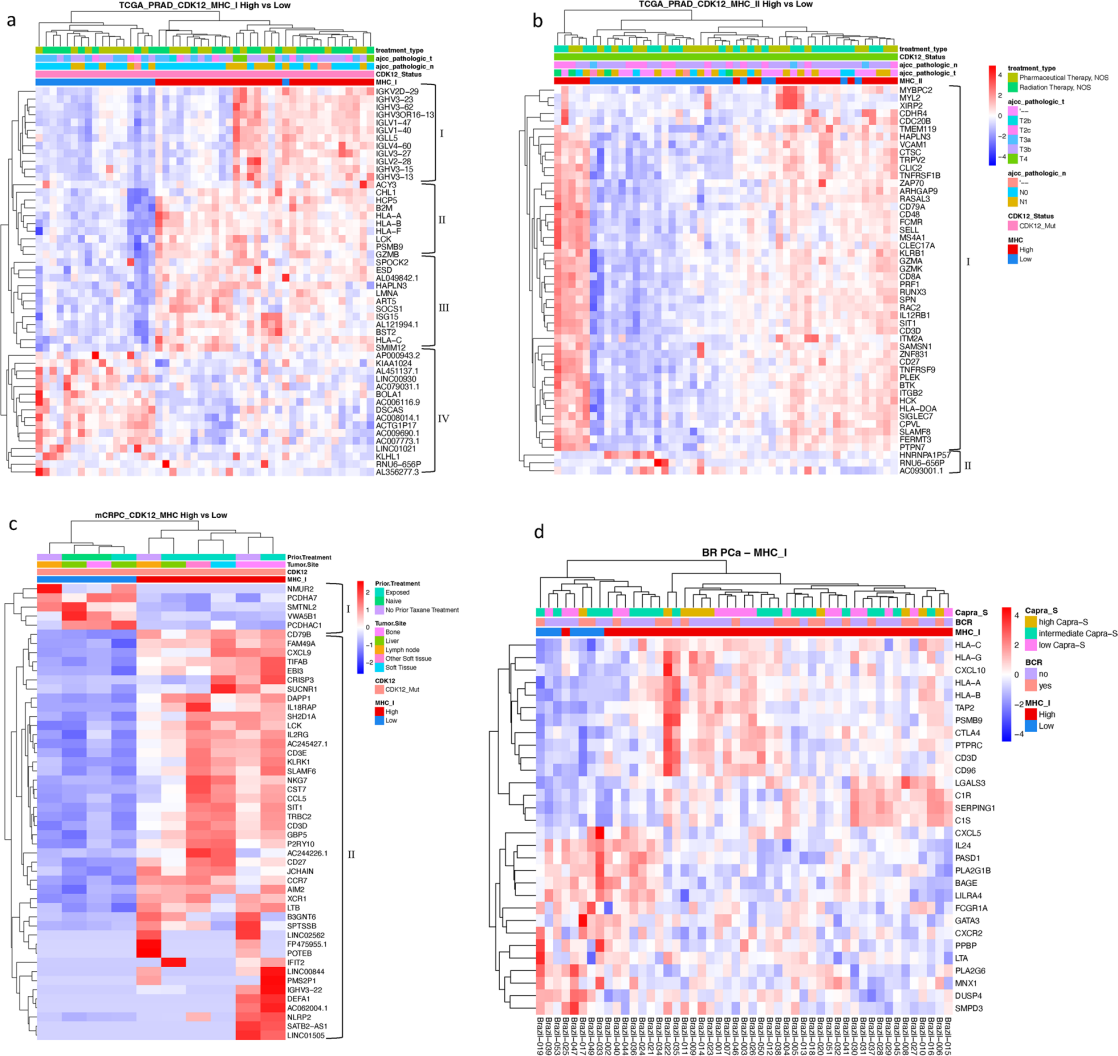
MHC-I/-II high expression in CDK12 defective prostate cancer showed distinct transcriptome activity. **a** Transcriptome heatmap exhibiting clustering of top 50 DEGs in pPCa CDK12-Mut MHC-I High group (*n* = 30). The upper cluster (I) has IGHV and IGLV over-expressed genes linked to *CDK12* MHC I high. The central clusters (II and III) have four HLA genes overexpressed in the MHC I high group, supporting our CDK12-mutated MHC expression classification. The lower cluster (IV) has 14 downregulated genes when MHC I is highly expressed. **b** Transcriptome heatmap exhibiting clustering of top 50 DEGs in the pPCa CDK12-Mut MHC-II High group (*n* = 23). The upper cluster (I) has upregulation of genes related to cytotoxicity, immune cell migration, and immune suppression. We could not observe clinical features associated with MHC I or II clusters. **c** Transcriptome heatmap exhibiting clustering of top 50 DEGs in the mCRPC CDK12-Mut MHC High group (*n* = 6). The combined MHC I and II showed two distinct clusters. The lower cluster showed the upregulation of genes linked to innate and adaptive immune response and CD4 + and CD8 + T cell activity. **d** Transcriptome heatmap exhibiting clustering of the DEGs from our validation cohort (FMRP). The DEGs are relative to the MHC-low ‘Low’ group. Clinical information is displayed on top of the heatmap for each patient. The color scale in the heatmap represents the Z-score of the normalized read counts for each gene, where the red scale indicates upregulated and blue low-expressed genes

Our hypothesis centers around the potential utility of classifying the *CDK12*-mut PCa based on their varying levels of MHC expression, offering additional information on immune response pathways and the TME for this molecular subtype of PCa. We compared the transcriptomics of *CDK12*-mut classified as “MHC high” (top 75% quartile of MHC expression) to the transcriptomics of tumors classified as having a low expression of MHC genes (bottom 25% quartile). Among the 48 pPCa *CDK12*-mut tumors, the group with MHC-I high expression showed 504 DEGs (Additional files 5, 6: Tables S4 and S5) compared to the MHC-I low group and showed four apparent clusters amongst the top DEGs (Fig. 1a). The upper cluster (I) demonstrates *IGHV* and *IGLV* over-expressed genes linked to *CDK12*-mut MHC-I high. The central clusters (II, III) have four *HLA* genes overexpressed in the MHC-I high group, which supports our classification based on *CDK12*-mut and MHC expression (*e.g.*, *HLA-A*, *HLA-B*, *HLA-C*). Also, this cluster showed upregulation of many genes related to antigen presentation and CD8 + T cell activity (Fig. 1a). The lower cluster (IV) possesses 14 downregulated genes when MHC-I is highly expressed. Similarly, *CDK12*-mut MHC-II high exhibited 503 DEGs compared to the MHC-II low group (Additional files 5, 6: Tables S4 and S6). Our comparison showed two clusters in the top DEGs (Fig. 1b). The upper cluster showed the upregulation of genes related to cytotoxicity, immune cell migration, and immune suppression (Fig. 1b). Our analysis of the mCRPC tumors identified 240 DEGs in *CDK12*-mut MHC-high (Additional file 5, 8: Tables S4 and S7) in comparison to the low group. The two clusters from the top DEGs showed the upregulation of genes linked to innate and adaptive immune response CD4 + and CD8 + T cell activity (Fig. 1c).

To validate these public domain transcriptomic data, we classified gene expression from our institutional FMRP cohort of pPCa transcriptome data for MHC genes using the same quartile analysis of DEGs described above. The comparison between MHC-high and MHC-low expressed groups identified 30 DEGs (Fig. 1d). Within the two observed clusters, the patients classified as MHC-I high group exhibited overexpression of *HLA* genes, consistent with our classification based on our in silico interrogation of the RNA-seq data in both public domain samples. Additionally, several genes were commonly expressed, including *CTLA4* and *HLA-G* (Additional file 9: Table S8). In general, for both pPCa and mCRPC cancers, *CDK12* -mut tumors expressing higher MHC levels were associated with transcriptomic changes that indicate a general pattern of activation of IFN-γ-responsive and cytotoxic activity genes.

**Fig. 2:**
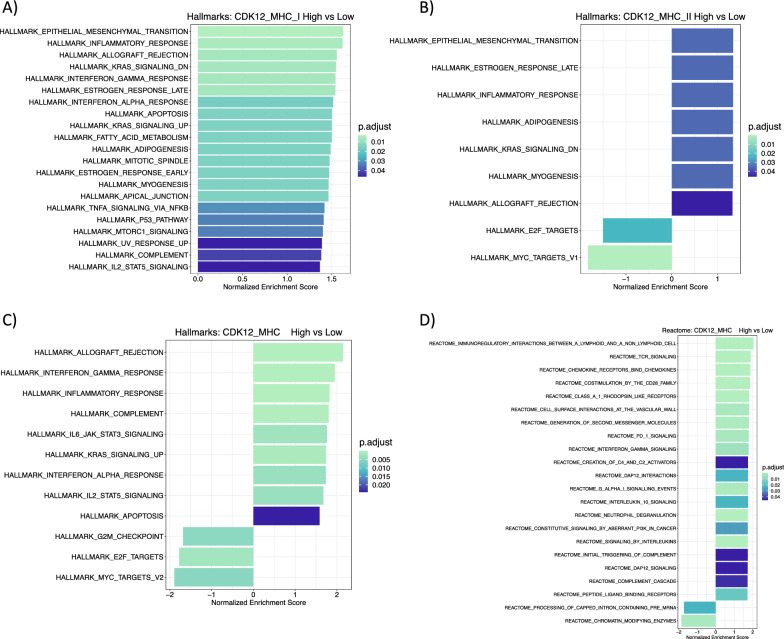
MHC high expressed in *CDK12*-altered prostate tumors are associated with the activation of immune-related pathways. Gene Set Enrichment Analysis (GSEA) of Hallmark pathways in MHC-I High pPCa **(a)** (*n* = 30), in MHC-II High pPCa **(b)** (*n* = 23), and of Hallmarks and Reactome pathways in MHC High mCRPC (*n* = 10) **(c, d)**. A normalized enrichment score indicates each factor’s positive or negative association with the condition of interest, which means activation or suppression of the pathway. Enrichment analyses were performed using *clusterProfiler* and *P*-adjusted value = 0.05 as the cutoff. Enrichment scores for each comparison are described in Additional file 6: Tables S5 (MHC-I High pPCa), S6 (MHC-II High pPCa), and S7 (MHC-I High mCRPC)

To better understand the transcriptional alterations in *CDK12*-mut tumors classified by variable expression of MHC-I/-II, we used enrichment analysis to identify functional associations of the DEGs with ICB response in the public domain data*.* In pPCa cases, *CDK12*-mut tumors with MHC-I and MHC-II high-expressed profiles showed significative enrichment of pathways related to activation of immune cells and antigen presentation, as expected (Table 1, Additional file 5, 6: Tables S4 and S5) consistent with an inflamed, or active TME phenotype. Furthermore, these groups showed expression of many pathways related to immune activation using GSEA analysis (*e.g.*, Interferon Gamma Response, Interferon Alpha Response, TNFA Signaling via NFKB, IL2/STAT5 Signaling, and Allograft Rejection (Fig. 2a, b, Additional file 6, 7: Tables S5 and S6). The DEGs from mCRPC *CDK12*-mut MHC high expressed cases showed enrichment of cytotoxicity and adaptive cytotoxicity immune response (Table 1, Additional file 8: Table S7). In addition, GSEA results showed activation of various hallmark and Reactome pathways (*e.g.*, Allograft Rejection, Interferon Gamma Response, Interferon Alpha Response, IL2/STAT5 Signaling, TCR Signaling, Interleukin 10 Signaling, and the suppressive PD1 Signaling pathway) (Fig. 2c, d, Additional file 8: Table S7). Enrichment analysis in our validation FMRP cohort showed common activation of several immune-related pathways. These findings demonstrate that our classification based on variation in MHC expression can identify tumors with various pathways associated with an active and inflamed TME in both cohorts. Furthermore, these results provide evidence of the feasibility of using MHC expression levels to subclassify the *CDK12*-mut patients into tumors that are more- or less likely to have activated immune evasion mechanisms.

**Table 1:** Enrichment results

	ID	Description	*P*-adj
TCGA_MHC-I	GO:0051249	Regulation of lymphocyte activation	1.48E−19
	GO:0002460	Adaptive immune response based on somatic recombination of immune receptors built from immunoglobulin superfamily domains	1.48E−19
	GO:0050867	Positive regulation of cell activation	2.78E−20
	GO:0002449	Lymphocyte mediated immunity	1.16 E−18
	GO:0006958	Complement activation, classical pathway	2.77 E−14
	GO:0006959	Humoral immune response	1.08 E−09
	GO:0050853	B cell receptor signaling pathway	2.20 E−08
	GO:0002440	Production of molecular mediator of immune response	7.15 E−08
	GO:0042742	Defense response to bacterium	1.39 E−07
	GO:0006910	Phagocytosis, recognition	5.66 E−08
TCGA _MHC-II	GO:0051249	Regulation of lymphocyte activation	2.00 E−25
	GO:0042110	T cell activation	5.84 E−25
	GO:0050867	Positive regulation of cell activation	9.60 E−23
	GO:0007159	Leukocyte cell–cell adhesion	2.50 E−20
	GO:0002460	Adaptive immune response based on somatic recombination of immune receptors built from immunoglobulin superfamily domains	2.80 E−20
	GO:0002443	Leukocyte mediated immunity	3.53 E−19
	GO:1903037	Regulation of leukocyte cell–cell adhesion	8.17 E−19
	GO:0070661	Leukocyte proliferation	1.75 E−15
	GO:0046651	Lymphocyte proliferation	5.77 E−14
	GO:0070663	Regulation of leukocyte proliferation	1.64 E−12
mCRPC_MHC	GO:0051251	Positive regulation of lymphocyte activation	4.87 E−15
	GO:0002449	Lymphocyte mediated immunity	6.43 E−15
	GO:0002253	Activation of immune response	6.67 E−15
	GO:0042110	T cell activation	3.27 E−11
	GO:0002460	Adaptive immune response based on somatic recombination of immune receptors built from immunoglobulin superfamily domains	1.76 E−09
	GO:0006959	Humoral immune response	1.21 E−06
	GO:0019724	B cell mediated immunity	2.55 E−06
	GO:0050863	Regulation of T cell activation	9.19 E−07
	GO:0007159	Leukocyte cell–cell adhesion	3.27 E−04
	GO:1903037	Regulation of leukocyte cell–cell adhesion	1.75 E−03
FMRP	GO:0019221	Cytokine-mediated signaling pathway	< 0.001
	GO:0022409	Positive regulation of cell–cell adhesion	< 0.001
	GO:0002474	Antigen processing and presentation of peptide antigen via MHC class I	< 0.001
	GO:0072182	Regulation of nephron tubule epithelial cell differentiation	< 0.001
	GO:0060333	Interferon-gamma-mediated signaling pathway	< 0.01
	GO:0002479	Antigen processing and presentation of exogenous peptide antigen via MHC class I, TAP-dependent	< 0.01
	GO:0042127	Regulation of cell population proliferation	< 0.01
	GO:0042590	Antigen processing and presentation of exogenous peptide antigen via MHC class I	< 0.01
	GO:0010719	Negative regulation of epithelial to mesenchymal transition	< 0.01
	GO:0048640	Negative regulation of developmental growth	< 0.01

Enriched GO (BP) pathways of the upregulated genes from TCGA *CDK12*-Mut MHC-I and MHC-II High, mCRPC group MHC High, and for our validation FMRP cohort MHC-I High group. Enrichment analyses were performed using *clusterProfiler* and *P*-adjusted value = 0.05 as the cutoff

### CDK12-mut MHC-I/-II high groups are associated with a tumor microenvironment with high expression of chemokines and immunomodulatory genes

**Fig. 3:**
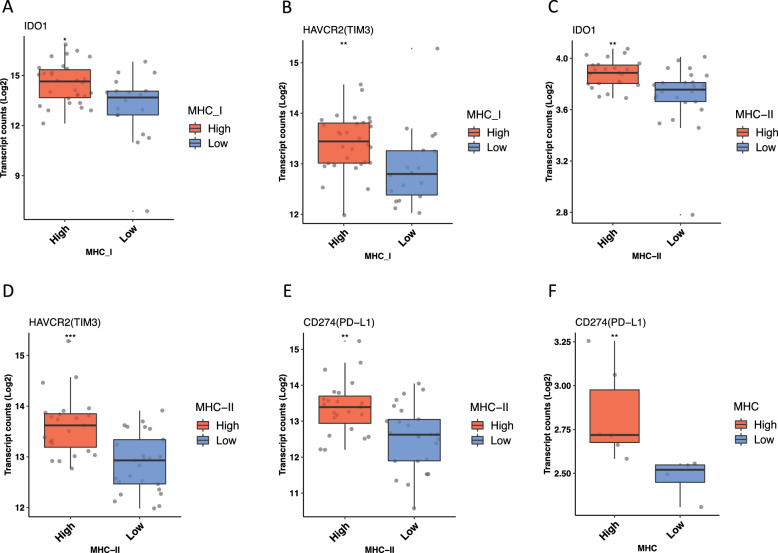
MHC high expressed CDK12 defective prostate cancer showed enhanced expression of immunomodulatory genes. Normalized RNA-seq expression level of the following genes was found to be significant higher: **a** *IDO1* (*P* < 0.018), **b** *HAVCR2* (*TIM3*) (*P* < 0.0022) in *CDK12-*Mut MHC-I High (*n* = 48); **c** *CD274* (*PD-L1*) (*P* < 0.0028), (d) *HAVCR2* (*TIM3*) (*P* < 0.00012)*,* (e) *IDO1* (*P* < 0.001) in *CDK12-*Mut MHC-II High (*n* = 48); and (f) *CD274* (*PD-L1*) (*P* < 0.0095) (*n* = 10). The statistical analysis revealed that p-values were less than 0.05 (*), and 0.01(**), respectively, as determined by the Mann–Whitney test. *IDO1*, Indoleamine 2,3-Dioxygenase 1; *HAVCR3* (*TIM3*), T-Cell Immunoglobulin Mucin Receptor 3; *CD274* (*PD-L1*), Programmed Cell Death 1 Ligand 1. The analysis used the low MHC group (Blue) as a control

Using MHC genes, our classification identified *CDK12*-mut tumors with an active and inflamed TME, which is typically associated with a favorable response to immunotherapy. However, chronic inflammation can also activate immunomodulatory mechanisms and immune checkpoint proteins potentially leading to resistance to ICB. In the public domain pPCa cohort, the *CDK12*-mut MHC-I/II high groups exhibited upregulation of many chemokines linked to APCs and effector T cell migration and the immunomodulatory genes *HAVCR2* (*TIM3*)*, IDO1*, and *CD274* (*PD-L1*) (Fig. 3a–e). Correlation analysis showed a significant positive correlation between MHC-I complex genes and *IDO1* but with no other investigated gene (Pearson Correlation, *p* < 0.05; Addition file 1, Figure S2a). Interestingly, among the MHC-II genes, correlation analysis showed a significant positive association between the expression of the MHC-II complex and the immunomodulatory genes *CD274* (*PD-L1*), *IDO1*, and *HAVCR2* (*TIM3*) (Pearson Correlation, *p* < 0.05, Figure S2b). The *CDK12*-mut mCRPC MHC high group exhibited high expression of chemokines and the immunomodulatory gene *CD274* (*PD-L1*) (Fig. 3f). Furthermore, we observed a significant positive association between the immunomodulatory genes *HAVCR2* (*TIM3*) and *CTLA4*, *LAG3*, and MHC-I and -II genes (Addition file 1, Figure S2c, d). Among *CDK12* defective tumors with higher expression levels of MHC genes, a pattern of upregulation of chemokine and immunomodulatory mechanisms was shown and are consistent with an active and inflamed TME phenotype. In contrast, *CDK12*-mut patients with low MHC expression exhibits alternative expression of immunomodulatory mechanisms associated with a cold TME phenotype and immune evasion.

### CDK12-mut MHC-I/-II high tumors have distinct immune cell infiltrate in the TME

**Fig. 4:**
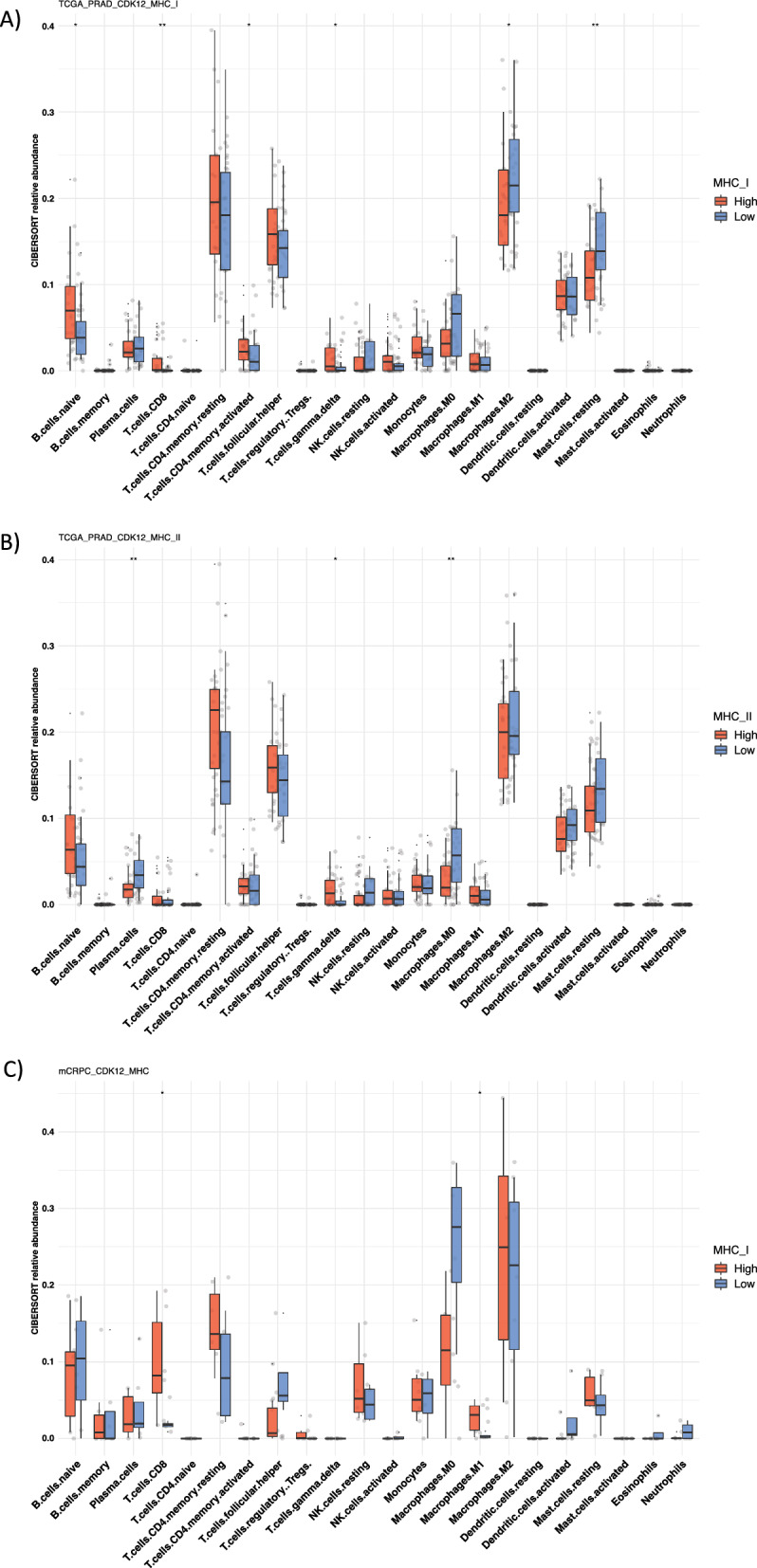
MHC high expressed *CDK12* defective is associated with enhanced T cell recruitment of prostate tumors. CIBERSORT-derived immune cell abundance of 22 cell subsets was found to be significant in **a** MHC-I High pPCa (*n* = 30), **b** MHC-II High pPCa (*n* = 23), and **c** MHC High mCRPC (*n* = 10). The statistical analysis revealed that p-values were less than 0.05 (*), and 0.01(**), respectively, as determined by the Mann–Whitney test. The analysis used the low MHC group (Blue) as a control

The active and inflamed TME phenotype in *CDK12*-mut patients with high MHC expression observed in the public domain cohort predicted that these tumors possess enrichment of immune cells with high effector and cytotoxic activity and the co-inhibitory expression of inhibitory pathways (Figs. 1, 2, 3). To test this hypothesis, we used in silico cytometry (CIBERSORTx) to estimate the immune cell composition in the TME of these tumors. The same comparison between *CDK12*-mut tumors expressing high vs. low MHC levels was similarly performed in the samples from the two public domain cohorts (TCGA-PRAD and mCRPC). In the pPCa cohort, *CDK12*-mut MHC-I high cases exhibited a significant increase in the composition of Naïve B cells, CD8 + T cells, and γδ T cells, and reduced infiltration of Mastocytes (Fig. 4a). In comparison, tumors with MHC-II high expressed shown an increase of γδ T cells and reduced composition of Plasma cells and M0 macrophages (Fig. 4b). The mCRPC *CDK12*-mut MHC high cases showed a significative increased composition of CD8 + T cell and M1 Macrophages (Fig. 4c). These results suggest that *CDK12*-mut patients with high MHC expression possess higher APCs and effector lymphocyte traffic in their TME compared to the lower MHC expression group. Furthermore, this demonstrates that the classification using MHC genes can predicted that these tumors possess high effector and cytotoxic immune cell abundance in the TME, which is an important factor when considering ICB treatment and further response.

### Genomic alterations of in CDK12-mut associated with low MHC-I/-II expression

During tumor evolution, the infiltration of cytotoxic lymphocytes eliminates highly immunogenic tumor clones, causing a preferential selection and survival of cell populations that have acquired MHC alterations. To determine whether the low levels of MHC-I/-II genes in *CDK12*-mut tumors possessed somatic genomic alterations affecting antigen presentation genes, we used an allele-specific copy number algorithm to estimate the copy number profile of *CDK12*-mut tumors expressing low levels of MHC-I/-II genes. The algorithm incorporates quantitative analysis of DNA data derived from WES, including MHC-I/-II and *HLA* genes on chromosome 6 and genes such as *PTEN*, commonly subject to chromosome 10 deletion and LOH in PCa [60]. Estimates include the determination of the relative clonality of allele-specific copy number alterations based on the fraction of tumor cells bearing LOH.

WES of pPCa revealed both subclonal allele-specific copy-neutral losses of heterozygosity (CN-LOH) and complete LOH events affecting MHC-I/-II and *HLA* genes on chromosome 6. Both major clonal and minor subclonal events were detected by comparing the copy number to the estimated cellular fraction of tumor cells harboring the copy number alteration (Table 2 and Figure S3). One example is illustrated by tumor TCGA-KK-A8IA in which only one major clonal event was detected with a cellular fraction = 0.833, and LOH events were detected on chromosomes 2, 5, and 10. While TCGA-YL-A8HO revealed four subclonal events, capturing biallelic loss, CN-LOH, and LOH in chromosomes 2, 5, 6, 15, and 16. Tumors expressing lower levels of MHC-I/II genes may be associated with the somatic genomic LOH events affecting regional transcriptional regulators of MHC expression and the cis-acting regulatory components of the MHC (Table 2). Two patients also showed subclonal complete loss of the *B2M* locus (ID: TCGA-EJ-8471, cellular fraction = 0.37; TCGA-YL-A8HO, cellular fraction = 0.27. Table 2), an important component of the MHC-I [61]. In contrast to pPCa, metastatic tumors harboring *CDK12* alterations expressing low levels of MHC-I/II showed allele-specific copy number gains in the MHC-I/I*I* complex and key regulators of MHC expression (Table 3 and Figure S4). Interestingly, one patient showed a clonal LOH event at the *JAK2* and *B2M* loci (ID: 5,115,615, cellular fraction = 0.90).

**Table 2:** Chromosomal CNA profiles in primary MHC low expressed tumors

Patient ID	Gene	Chrom	Start	End	Cellular fraction	Total copy number	Minor copy number
KK.A8IA	*STAT1*	2	134713128	192194260	0.833	1	0
KK.A8IA	*IRF1*	5	55231421	143147410	0.833	1	0
KK.A8IA	*PTEN*	10	87552401	89139399	0.833	1	0
KK.A8IA	*B2M*	15	41191425	77615398	0.208	0	0
KK.A8IG	*IFNGR1*	6	65302925	170584366	0.668	1	0
KK.A8I6	*PTEN*	10	73243437	104454566	0.332	0	0
KK.A8I6	*NLRC5*	16	49396608	90096324	0.11	0	0
XJ.A9DX	*NLRC5*	16	49280867	57722991	0.62	1	0
ZG.A9LM	*STAT1*	2	134317491	192057773	0.47	2	0
ZG.A9LM	*B2M*	15	19882284	101922415	0.47	2	0
ZG.A9LM	*NLRC5*	16	56276055	89228602	0.47	2	0
EJ.8472	*JAK1*	1	21619903	67705610	0.37	1	0
EJ.8472	*B2M*	15	42751857	97971259	0.37	2	0
XQ.A8TA	*IRF2*	4	177976002	189984351	0.92	1	0
XQ.A8TA	*IRF1*	5	128464930		0.92	2	0
XQ.A8TA	*PTEN*	10	87790275	89599433	0.92	0	0
YL.A8HO	*STAT1*	2	119157673	199659594	0.12	0	0
YL.A8HO	*IRF1*	5	143082	181260211	0.38	2	0
YL.A8HO	*MHC-I/II, TAP1/2*	6	304637	170584276	0.27	1	0
YL.A8HO	*IFNGR1*	6	304637	170584276	0.27	1	0
YL.A8HO	*B2M*	15	19882294	101922415	0.27	1	0
YL.A8HO	*CIITA*	16	8644541	58196892	0.22	2	0
YL.A8HO	*NLRC5*	16	8644541	58196892	0.22	2	0
XK.AAJA	*PTEN*	10	7243639	12235132	0.54	1	0
KK.A7AP	*B2M*	15	33311111	57091863	0.87	1	0
KK.A7AP	*NLRC5*	16	48347484	90177833	0.87	1	0
HC.7744	*B2M*	15	42515191	45178103	0.12	0	0
KK.A8IG	*IFNGR1*	6	65302925	170584366	0.668	1	0
EJ.7784	*JAK2*	9	14859	18718116	0.437	1	0
HC.A48F	*JAK1*	1	41513578	80451865	0.86	1	0
HC.A48F	*IRF2*	4	85671	189984257	0.21	1	0
HC.A48F	*IRF1*	5	54400082	171456817	0.86	1	0
HC.A48F	*MHC-I/II, TAP1/2*	6	304637	170584366	0.31	1	0
HC.A48F	*IFNGR1*	6	304637	170584366	0.31	1	0
HC.A48F	*PTEN*	10	49168049	118015066	0.21	1	0
HC.A48F	*B2M*	15	44380814	50749712	0.86	1	0
HC.A48F	*NLRC5*	16	35085998	90177833	0.86	1	0

Integral results of FACETS analysis of whole-exome sequencing data from *CDK12* defective primary tumors expressing low levels of MHC-I/II. The table shows the integer chromosomal copy numbers for the allelic ratio of the heterozygous SNPs in each patient's tumor/normal pair. The first column is the patient ID, and the second column is the MHC region loci and other genes commonly subject to LOH in PCa. The last two columns represent the total copy number and the minor copy number, respectively. Tumors with a 2:1 ratio are considered normal for each position, while 2:0 and 1:0 represent CN-LOH and LOH events. The cellular fraction represents the estimated number of cells harboring the genotype. Primary PCa revealed subclonal CN-LOH and LOH at *JAK1, STAT1, IRF2, IRF1, MHC-I/II, TAP1, TAP2, IFNR1, JAK2, B2M, CIITA, NLRC5,* and *IFNR1*. Two patients showed biallelic loss at the *B2M* locus (Total copy number/minor copy number ratio = 0:0). *MHC-I = *HLA-A, HLA-B,* and *HLA-C;* MHC-II = *HLA-DRA, HLA-DRB5, HLA-DRB6, HLA-DPA1, HLA-DPB1, HLA-DQA1, HLA-DQB1,* and *HLA-DQB2*

**Table 3:** Genomic profile in metastatic MHC low expressed tumors

Patient ID	Gene	Chrom	Start	End	Cellular fraction	Total copy number	Minor copy number
5115412	*JAK1*	1	33804897	68440771	0.49	5	2
5115412	*STAT1*	2	187346385	205615949	0.54	5	2
5115412	*IRF2*	4	182891198	185757023	0.87	6	2
5115412	*IRF1*	5	112892242	139881181	0.81	4	2
5115412	*MHC-I*	6	29555893	32256053	0.87	4	1
5115412	*MHC-II*	6	32288409	38345160	0.30	6	2
5115412	*TAP-1*	6	32288409	38345160	0.30	6	2
5115412	*TAP-2*	6	32288409	38345160	0.30	6	2
5115412	*JAK2*	9	2804198	15486990	0.59	4	2
5115412	*PTEN*	10	80253502	93958843	0.30	6	2
5115412	*B2M*	15	30626570	59138344	0.59	4	2
5115412	*CIITA*	16	198972	17358048	0.59	4	2
5115412	*NLRC5*	16	57372390	71634040	0.52	4	2
5115412	*IFGRR2*	21	33230307	34889712	0.87	5	2
1115161	*IRF2*	4	184116877	186709443	0.51	6	3
1115161	*HLA-A*	6	29330997	30628358	0.79	3	1
1115161	*HLA-B*	6	30635016	31382266	0.59	5	2
1115161	*HLA-C*	6	30635016	31382266	0.59	5	2
1115161	*JAK2*	9	52538	33113788	0.20	3	1
5115615	*JAK1*	1	63571201	65432064	0.912	3	1
5115615	*STAT1*	2	188522965	192194738	0.912	3	1
5115615	*MHC-I*	6	29944749	30415099	0.899	3	1
5115615	*MHC-I/II*	6	30469283	32407241	0.902	4	2
5115615	*MHC-II*	6	32439760	32666486	0.912	3	1
5115615	*MHC-II/TAP1/2*	6	32666737	34289022	0.902	4	2
5115615	*JAK2*	9	117666	9091026	0.906	1	0
5115615	*B2M*	15	41691484	46828068	0.906	1	0
5115615	*CIITA*	16	9938304	11178578	0.912	3	1
5115615	*IFGRR2*	21	33335636	37018290	0.899	3	1
5115412	*JAK1*	1	33804897	68440771	0.49	5	2
5115412	*STAT1*	2	187346385	205615949	0.54	5	2
5115412	*IRF2*	4	182891198	185757023	0.87	6	2
5115412	*IRF1*	5	112892242	139881181	0.81	4	2
5115412	*MHC-I*	6	29555893	32256053	0.87	4	1
5115412	*MHC-II*	6	32288409	38345160	0.30	6	2
5115412	*TAP-1*	6	32288409	38345160	0.30	6	2
5115412	*TAP-2*	6	32288409	38345160	0.30	6	2

Integral results of FACETS analysis of whole-exome sequencing data from *CDK12* defective metastatic tumors expressing low levels of MHC-I/II. The same parameters shown in Table 1 were used to characterize LOH for each gene of interest. Metastatic PCa showed allele-specific copy number gains at *JAK1, STAT1, IRF2, IRF1, MHC-I/II, TAP1, TAP2, IFNR1, JAK2, B2M, CIITA, NLRC5,* and *IFNR1*. One patient showed a clonal LOH event at the *JAK2* and *B2M* loci (Total copy number/minor copy number ratio = 1:0). *MHC-I = *HLA-A, HLA-B,* and *HLA-C;* MHC-II = *HLA-DRA, HLA-DRB5, HLA-DRB6, HLA-DPA1, HLA-DPB1, HLA-DQA1, HLA-DQB1,* and *HLA-DQB2*

We utilized the Oncomine Assay Plus (OCA-Plus, Thermofisher) to detect *CDK12* and other mutations in our validation FMRP cohort. Out of 53 sequenced patients, two patients (17 and 38) exhibited *CDK12* (4%) mutations (SNVs are indicated in Additional file 4: Table S3), and we performed additional Sanger sequencing to validate these results. Only patient 17 demonstrated positive signals compared to our control samples (Additional file 1: Figs. S5 and S6). No other genomic, SNV, LOH or copy number gains events affecting regional transcriptional regulators of MHC expression, and the cis-acting regulatory components of the MHC allele-specific were identified, although this patient was classified as MHC low (Fig. 1d).

Our combined analysis of *CDK12*-defective public domain data and our cohort study indicate that low expression of MHC in primary tumor clones are often linked to structural alterations, such as somatic CN-LOH and LOH subclonal genomic alterations. The presence of subclonal LOH suggests that ongoing selective processes may favor genomic mechanisms leading to reduction in MHC expression. In contrast, metastatic tumors revealed both LOH and high copy-number gains affecting antigen presentation genes and their regulators. Therefore, these subclonal regulatory mechanisms, including CN-LOH and LOH events, are associated with reduced MHC expression that may contribute to impaired tumor immunogenicity.

## Discussion

*CDK12* inactivation is known to increase the immunogenicity of tumor cells [7, 14, 62–64]. MHC-I and -II expression changes are involved in tumor immune evasion in various cancer types [44, 48, 65, 66]. In samples from public domain cohorts, we observed two groups of *CDK12*-mut tumors with respect to MHC expression. Further classification using MHC genes and in silico analysis of RNA-seq indicated that higher levels of MHC are linked to the activation of multiple pathways associated with the immune system, significantly high expression of immunomodulatory genes*,* and increased CD8 + T cells, B cells, γδ T cells, and M1 Macrophages composition consistent with an inflamed TME. In contrast, lower MHC expression was associated with features related to an immunologically cold TME. Genomic analysis indicated that tumors with low MHC expression also exhibited allele-specific copy-number alteration in genes involved in regulating MHC expression and antigen presentation. Using an independent cohort of pPCa from our Institute, we validated that our classification based on MHC gene expression can identify tumors with various pathways associated with an active and inflamed TME.

Transcriptomic signatures associated with MHC expression variation have been described in different tumor types [44]. These signatures capture the activity of genes and biological pathways related to tumor cells' crosstalk with the TME and appear to correlate with clinical responses to ICB [27, 44, 67–69]. Ayers et al*.* proposed a gene expression profile with eighteen genes relevant to predicting the clinical outcome of anti-PD1 therapy [67]. This IFN*-*γ gene signature in pretreatment tumor biopsies was associated with improved outcomes in melanoma, head-and-neck squamous cell carcinoma, and gastric cancer treated with pembrolizumab. In our study, the *CDK12* patient tumors expressing high levels of MHC genes showed the presence IFN*-*γ gene signature, which might reflect a better response to anti-PD1 therapy (Figure S7). Our results also showed the common activation of IFN*-*γ response genes and pathways in both the public domain samples and independent cohort (Figs. 1, 3, 4b, c).

An IFN*-*γ gene signature can alternatively activate the expression of immunomodulatory mechanisms and promote adaptive resistance to ICBs such as anti-PD1 [70, 71]. IFN*-*γ is a key player in the elimination phase during immunoediting [72]. We identified the upregulation of immunomodulatory molecules, such as *IDO1*, *TIM3,* and *CD274(PD-L1),* in both prostate tumors expressing higher levels of MHC-I and -II genes (Fig. 3). Interestingly, the *CDK12* patient tumors expressing high levels of MHC genes showed the IFN-γ gene signature, including upregulation of a non-classical MHC molecule HLA-E [73, 74]. We also found a link between *IGHV* and *IGLV* over-expressed genes in a group of the *CDK12*-mut pPCa expressing high levels of MHC-I (Fig. 1a, upper cluster I). The production of Ig by tumor cells (cancer-derived Ig) is described in various cancers and PCa [73–76]. Also, cancer-derived Ig may act as checkpoint proteins and inhibit effector T cells and NK cells [74, 77].

The response to exacerbated IFN-γ is also associated with the development of protumor molecular mechanisms leading to an immunosuppressive and tolerogenic TME [70]. IFN-γ is known to induce the expression of suppressive molecules such as IDO1, and HLA-E, which are known regulators of CTL and NK cells [72, 73]. The IFN-γ signaling process can impair the body's antitumor immunity by triggering a feedback loop that weakens it. For example, this feedback loop can be activated by the PD-1 signaling pathway, which is directly upregulated by IFN-γ signaling. The ligands PD-L1 and PD-L2 are then upregulated in tumor, stromal, and immune infiltrate cells, and these ligands interact with PD-1 on tumor-infiltrating T cells, causing a decrease in their cytotoxic response [78, 79]. Our findings indicate the presence of acquired somatic chromosomal resistance mechanisms such as LOH of MHC impairing the expression of MHC genes and that CDK12-mut tumors with normal expression of these genes display expression of suppressive molecules in response to exacerbate IFN*-*γ. These findings suggest that measuring the basal expression of MHC could be used to further characterize *CDK12* defective tumors, providing insights as to how the expression of immunomodulatory genes might be associated with resistance to ICB.

The expression of MHC molecules plays a pivotal role in providing the signals necessary to recognize and activate the immune system against tumor neoantigens and is essential to controlling tumor growth through cytotoxic activity [72, 80]. We found a higher abundance of CD8 + T cells in *CDK12*-mut tumors expressing high levels of MHC genes (Fig. 4). This result may either indicate the dependence of CD8 + T cells in MHC-I antigen presentation or suggest that enhanced CD4 + Th infiltrate could support the continued accumulation of CD8 + CTLs in the TME [68]. Interestingly, *CDK12* pPCa with high expression of both MHC-I and -II genes showed increased levels of γδ T cells (Fig. 4a, b). The basal effector immune cell population, such as γδ T cells in pPCa, may contribute to IFN*-*γ signaling and indicate the dependence of the MHC-unrestricted recognition role of γδ T cells and its presence in the TME [27, 81].

The loss of MHC expression could be derived from two different types of disruption [82]. First, regulatory abnormalities downregulate the expression of MHC genes through mechanisms that do not affect the genomic structure of HLA genes (“Soft” lesions). In such cases, specific T-cells can recover the MHC expression-mediated response (e.g., IFN-γ signaling). Second, during tumor evolution, the infiltration of cytotoxic lymphocytes eliminates tumor clones with high immunogenicity, causing a selection of clones with reduced MHC expression that may present structural alteration, or “Hard” lesions, in the MHC loci or other genomic regions (e.g., *B2M*, *IFN*, *STAT*) distinct from the derived clone [61].

Somatic LOH affecting large genomic regions can lead to changes in gene expression through various mechanisms including loss of functional alleles, haploinsufficiency, disruption of regulatory elements, and alterations in epigenetic modifications during tumor progression [83]. These changes can have significant implications for anti-cancer immune responses if they confer a selective advantage for immune evasion. Chromosomal studies of chromosome 6 LOH in various cancers suggest reduced MHC expression is often associated with genetic and genomic aberrations that may result in reduced antigen presentation and, thus, facilitate immune evasion [22, 84]. In keeping with these data, our analysis of WES from public domain samples revealed CN-LOH, LOH, and copy numbers gains in *CDK12*-mut tumors expressing low levels of MHC-I/II genes at known regulators of MHC expression and the components of the MHC (Tables 2 and 3). Two pPCa patients also showed complete loss of the *B2M* locus (Chr15:41691484–46828068), while one mCRPC exhibited a LOH event at the *B2M,* an important component of the MHC-I complex, and mutations of this gene have previously been associated with ICB resistance [61]. In addition, two of 53 patients showed *CDK12* mutation in the FMRP cohort, although only one with further validated through Sanger sequencing (Patient 17, Additional file 1: Fig. S5). This patient was classified as MHC low expression, high CAPRA-S score, and was diagnosed with biochemical recurrence post radical prostatectomy after six months. However, more extensive studies will be required to determine whether reduced MHC expression is a general feature of primary PCa or is a specific feature of *CDK12* mutated tumors.

Although we described and validated that the classification based on the MHC gene expression can identify tumors with impacted pathways linked to the immune system, this study has some limitations. Firstly, the frequency of *CDK12* mutation is very low at 1–2% in primary tumors and 5–7% in advanced PCa [12, 14, 15], and cohorts that contain a higher number of patients harboring this mutation and both RNA-seq and genome data are rare. Future studies in many patients harboring *CDK12* mutations are needed to address potential statistical bias regarding our low number of patients. Secondly, we did not address the potential molecular mechanism underlying the alteration of the low expression of MHC in *CDK12*-mut PCa, as shown in our patient 17, including epigenetic alterations, miRNA activity, and potential influence of adjuvant therapies on MHC expression and immune TME dynamics. Further studies are needed to approach the causes of MHC disruption derived from “Soft” alterations that occur in *CDK12*-mut prostate cancer. Thirdly, although the samples were derived from tumor-enriched regions from biopsies, we could not establish a limit for the contribution of the *CDK12*-mut tumor cells and TME to the MHC expression since the RNA-seq relies on data from bulk tissue [85]. The relative expression of HLA-A and -B has been shown to correlate with proteins expressed on the cell surface [86, 87]. The HLA-A and -B genes also undergo complex processing dynamics, in which differences between pre-mRNA and mature mRNA, and are proportionally degraded within the cell, suggesting transcription regulation, and splicing to be the dominant regulatory step in HLA expression. Moreover, studies by McCutcheon J. [88] and Aguiar, V.R.C. [45] further support this correlation, emphasizing the intricate interplay between mRNA levels and surface expression of HLA-C proteins.

## Conclusions

We distinguished two subsets of *CDK12* tumors based on differential MHC expression levels. Our *in-silico* analysis of public domain data and validation in our FMRP institutional cohort suggest that *CDK12*-mut PCa expressing higher levels of classical MHC genes have an active and inflamed TME with elevated immunomodulatory pathway expression and increased presence of effector T cells consistent with a hot TME. In contrast, tumors with decreased MHC expression showed chromosomal copy-number alteration in genes that regulate MHC expression and antigen presentation associated with a cold TME. More extensive in vitro and in vivo investigations are required to relate these two distinct subsets of *CDK12-*mut PCa to potential actionable immunomodulatory mechanisms and future therapeutic approaches. Depending on the mechanism, the downregulation of MHC expression can sometimes be therapeutically restored to improve anti-tumor immunity [89].

A recent report by Bergom et al. indicated that a tumor displaying high microsatellite instability and a deficiency in mismatch repair exhibited "cold" and "hot" tumor nodes with distinct TME. WES analysis revealed that the “cold” node was a subclone derived from the other node. Additionally, transcriptome analysis identified that the cold lesion had low expression of HLA genes, CTLA-4, and CD274, while the "hot" node was rich in CD8 + and CD4 + lymphocytes, γδ T cells, and NK cells [90]. These findings support our study and further reinforce the generalization of our hypothesis to high immunogenic PCa*.* The results suggest that MHC expression can be used to investigate those tumors that are more likely to respond to ICB treatment.

Future studies might explore using liquid biopsies in detecting *CDK12* mutations in PCa blood or urine, offering real-time information on mutation status, disease progression, and treatment response. Traditional IHC and advanced multiplexed immunofluorescence techniques can be used to validate and visualize the correlation between *CDK12* mutations and LOH-driven changes in MHC expression at the protein level in PCa specimens. Potential future treatments may involve agents that modulate the immune microenvironment, such as cytokines (e.g., interferons) and immune-stimulating compounds to enhance the immune response against *CDK12*-defective PCa. Also, combinatorial approaches involving traditional treatments, targeted therapies, and immunomodulation could be explored to maximize therapeutic outcomes.

### Supplementary Information

**Additional file 1. Supplementary figures. **This file contains supplementary figures related to the main analysis and results from the manuscript.

**Additional file 2. Table S1. **Databases and accession numbers. The original RNA-seq data were downloaded from the recount2 website (http://idies.jhu.edu/recount/data/fc_rc/rse_fc_TCGA_prostate.Rdata) and the database of Genomic and Phenotypes (dbGaP) under accession number phs000178.v11.p8.c1, phs000915.v2.p2.c1. The original clinical information of both cohorts is deposited in the CbioPortal (https://www.cbioportal.org) and GDCPortal (https://portal.gdc.cancer.gov)

**Additional file 3. Table S2. **Target genomic regions. This file highlights the genomic loci and associated genes studied using FACETS.

**Additional file 4. Table S3. **The primers and amplicon sequences used in PCR and Sanger Sequencing validation of CDK12 mutation in the FMRP cohort.

**Additional file 5. Table S4. **Summary results of DEGs primary (TCGA-PRAD) and advanced (mCRPC-SU2C) prostate cancer. (A) Number of DEGs in primary prostate tumors. (B) DEGs in metastatic castration-resistant prostate tumor.

**Additional file 6. Table S5. **Summary results of CDK12-mut TCGA-PRAD MHC-I high expressed.

**Additional file 7. Table S6. **Summary results of CDK12-mut TCGA-PRAD MHC-II high expressed.

**Additional file 8. Table S7. **Summary results of CDK12-mut mCRPC MHC high expressed.

**Additional file 9. Table S8. **Summary results of FMRP MHC high expressed.

## Data Availability

The original dataset is deposited in the database of Genomic and Phenotypes (dbGaP) under accession numbers *phs000178.v11.p8.c1*, *phs000915.v2.p2.c1.* The original clinical information of both cohorts is deposited in the CbioPortal and GDC Portal. All other data supporting the conclusions of this article are included within the article and its additional files. Additional files related to our retrospective cohort are available at GSE244631.
