# Supplementary material for: Loss of heterozygosity impacts MHC expression on the immune microenvironment in CDK12-mutated prostate cancer
Source: Mol Cytogenet. 2024 May 4;17:11. doi: 10.1186/s13039-024-00680-6 (PMC11070094; doi:10.1186/s13039-024-00680-6)
Supplement: Supplementary file 1 — Additional file 1. Supplementary figures. This file contains supplementary figures related to the main analysis and results from the manuscript. [file 13039_2024_680_MOESM1_ESM.docx]

Supplementary Material

**Figure S1. Classification of *CDK12* patients based on HLA genes (MHC-I and -II) expression.**

We observed two groups of *CDK12* defective tumors regarding MHC expression using hierarchical clustering analysis for pPCa and mCPRC. We then used the normalized expression level of each gene (e.g., MHC-I: *HLA-A*, *HLA-B*, *HLA-C*; MHC-II: *HLA-DPA1*, *HLA-DPB1*, *HLA-DQA1*, *HLA-DQB1*, *HLA-DQB2*, *HLA-DRA*, *HLA-DRB5*, *HLA-DRB6*) (Schaafsma et al. 2021) to classify the patients based on their MHC gene expression (See method). We dichotomized expression levels below or above the first quartile for each gene using the normalized read count; we then used this classification to generate the final logical values regarding the patient’s MHC status (‘High’ or ‘Low’ expressed) as follows: e.g., threshold *Gene 1* AND threshold *Gene 2* AND threshold *Gene 3*. Samples were classified as having MHC ‘Low’ expression when at least one gene composing each class was expressed at a low level. In mCRPC *CDK12*-mut, all tumors classified as MHC-High presented similar high expression of both MHC-I and -II; thus, we called these tumors just MHC High. The table above shows the final number of patients classified for each tumor type in each condition.

**Figure S2. Correlation of classical MHC and immunomodulatory genes.** (a-b) Correlation plots between *MHC-I /–II* and the immunomodulatory genes in primary prostate tumors. (c-d) Correlation plots between *MHC-I /–II* and the immunomodulatory genes in metastatic castration-resistant prostate tumors. Pearson Correlation was used in normalized expression levels of each gene (coef. level = 0.95). A significant correlation is indicated in nonmarket squares (*p*<0.05).

**Figure S3.** **Whole-genome sequencing data from *CDK12* defective in primary MHC low-expressed tumors.** Integrated visualization shows the total copy number log ratio (logR) on the top panel, and allele-specific log-odds-ratio data (logOR) on the second panel with chromosomes alternating in blue and gray. The third panel plots the corresponding integer (total, minor) copy number calls. Tumors with a 2:1 ratio are considered normal for each position, while 2:0 and 1:0 represent CN-LOH and LOH events. The estimated cellular fraction profile is plotted at the bottom, revealing both clonal and subclonal copy number events.

**Figure S4.** **Whole-genome sequencing data from *CDK12* defective in metastatic MHC low-expressed tumors.** Integrated visualization shows the total copy number log ratio (logR) on the top panel, and allele-specific log-odds-ratio data (logOR) on the second panel with chromosomes alternating in blue and gray. The third panel plots the corresponding integer (total, minor) copy number calls. Tumors with a 2:1 ratio are considered normal for each position, while 2:0 and 1:0 represent CN-LOH and LOH events. The estimated cellular fraction profile is plotted at the bottom, revealing both clonal and subclonal copy number events.

**Figure S5.** **BR PCa validation**

**Figure S6.** **BR PCa validation**

**Figure S7.** **Common activation of IFN-γ-responsive genes in *CDK12* defective MHC high expressed tumors.** In our study, the *CDK12* patient tumors classified as MHC High showed the presence of several genes previously described as IFN-γ-responsive and cytotoxic activity and associated with response to anti-PD1 therapy (Ayers, 2017. <https://doi.org/10.1172/JCI91190>). Common genes with Ayers et al. 2017, are indicated in the intersection and unique features for each comparison are indicated in red. Venn`s Diagrams by *Oliveros, J.C. (2007-2015) Venny. An interactive tool for comparing lists with Venn's diagrams.* <https://bioinfogp.cnb.csic.es/tools/venny/index.html>.
